# Transport capacity is uncoupled with endodormancy breaking in sweet cherry buds: physiological and molecular insights

**DOI:** 10.3389/fpls.2023.1240642

**Published:** 2023-11-14

**Authors:** Mathieu Fouché, Hélène Bonnet, Diane M. V. Bonnet, Bénédicte Wenden

**Affiliations:** INRAE, Univ. Bordeaux, UMR Biologie du Fruit et Pathologie 1332, Villenave d’Ornon, France

**Keywords:** bud dormancy, callose, *Prunus avium* L., temperature, transcriptomics, transport capacity

## Abstract

**Introduction:**

To avoid the negative impacts of winter unfavorable conditions for plant development, temperate trees enter a rest period called dormancy. Winter dormancy is a complex process that involves multiple signaling pathways and previous studies have suggested that transport capacity between cells and between the buds and the twig may regulate the progression throughout dormancy stages. However, the dynamics and molecular actors involved in this regulation are still poorly described in fruit trees.

**Methods:**

Here, in order to validate the hypothesis that transport capacity regulates dormancy progression in fruit trees, we combined physiological, imaging and transcriptomic approaches to characterize molecular pathways and transport capacity during dormancy in sweet cherry (Prunus avium L.) flower buds.

**Results:**

Our results show that transport capacity is reduced during dormancy and could be regulated by environmental signals. Moreover, we demonstrate that dormancy release is not synchronized with the transport capacity resumption but occurs when the bud is capable of growth under the influence of warmer temperatures. We highlight key genes involved in transport capacity during dormancy.

**Discussion:**

Based on long-term observations conducted during six winter seasons, we propose hypotheses on the environmental and molecular regulation of transport capacity, in relation to dormancy and growth resumption in sweet cherry.

## Introduction

Temperate perennials plants have built strategies to avoid the negative impacts of winter unfavorable conditions for plant development. At the beginning of autumn, temperate trees enter a rest period called dormancy. This process is necessary to stop growth and protect organs until favorable conditions resume in the spring. In fruit trees, this wintering period is essential in the seasonal cycle to ensure an abundant and qualitative blooming leading to the reproductive success and in consequence fruit production (Coville, 1920; Couvillon and Erez, 1985). In the recent decades, due to climate change, mild temperatures during winter have been more frequent, with potential impacts on the progression of bud dormancy (Campoy et al., 2011; Atkinson et al., 2013; Legave et al., 2015; Maurya and Bhalerao, 2017). For instance, it has been observed that flowering dates have significantly advanced in the last decades for several European species, and consequently could increase the risk of damages by late spring frost (Jochner et al., 2016). This potential issue must be considered as a serious threat and it could have an economic consequence on the forest and fruit tree industries (Warmund et al., 2008; Jochner et al., 2016). In order to anticipate climate change effects on dormancy, improving the knowledge on physiological and molecular mechanisms that regulate dormancy progression is necessary to propose solutions for cultivars better adapted to climate change.

Winter dormancy is a complex process that involves multiple signaling pathways (Horvath, 2009; Beauvieux et al., 2018; Fadón et al., 2020; Yang, 2021), when trees go through successive dormancy stages from the end of summer to spring, characterized by the tree’s capacity to respond to cold and mild temperatures (Lang et al., 1987). Previous studies have suggested that pathways involved in transport capacity may regulate the progression throughout these dormancy stages (Rinne et al., 2011; Tylewicz et al., 2018; Tixier et al., 2019; Savage and Chuine, 2021). Four main transport pathways have been described as potentially involved in the connection between buds and the twig, as well as within the bud itself: two vascular (the phloem and xylem systems) and two nonvascular (the apoplostatic pathway, which is associated to cell wall interspace and is continuous between xylem and the cell wall space, and the symplasmic pathway which corresponds to cell-to-cell transport via plasmodesmata, and is continuous between the phloem and all plasmodesmata linked-cells). Functions for all these pathways were shown to decrease during dormancy onset and are very limited during endodormancy, a deep dormancy phase triggered by low temperatures, short photoperiod and endogenous inhibitors, when buds cannot grow until a certain amount of cold is accumulated, defined as chill requirements. After chill requirements are satisfied, buds enter the ecodormancy stage, followed by growth resumption under favourable conditions, which is associated with a reopening of some transport pathways (Savage and Chuine, 2021). In more details, xylem continuity between twig and buds during dormancy could indeed be disrupted in order to prevent ice formation as a protection against frost (Ashworth, 1984; Sperry, 1993; Ameglio et al., 2002), potentially due to the presence of immature xylem cells or modifications to cell walls or tannin like substance (Goodwin, 1967), as shown in grape (Signorelli et al., 2020) and in pine trees (Lapa et al., 2017). However, recent studies have suggested that the symplasmic pathway may have the primary role in the dynamics of transport capacity throughout dormancy in trees (Rinne et al., 2011; Tylewicz et al., 2018). Short days were shown to promote dormancy onset of vegetative buds in hybrid aspen (*Populus tremula* × *P. tremuloides*) through the action of phytohormones, abscisic acid (ABA) and gibberellins (GA), associated with the blockage of inter-cellular communication through plasmodesmata (Tylewicz et al., 2018; Veerabagu et al., 2023). In this context, plasmodesmata closure is essential to maintain dormancy during winter, by preventing the circulation and response to growth promoting signals (Rinne and Schoot, 2003). This regulatory mechanism is mediated by callose, a β-1,3 glucan polysaccharide, that accumulates in cell walls around the plasmodesmata and control their permeability (Wu et al., 2018). Consistently, studies showed that dormancy induction and maintenance were promoted by a complex regulatory network involving genes related to plasmodesmata closure such as *CALLOSE SYNTHASE 1* (*CALS1*) (Maurya et al., 2018; Tylewicz et al., 2018; Zhang et al., 2018a; Singh et al., 2019; Singh et al., 2021). Chilling accumulation then induces dormancy release, which coincides with the expression of GA-inducible β-1,3 glucanases (*GH17* genes) leading to callose degradation and allowing the restoration of cell-to-cell communication (Rinne et al., 2001; Rinne et al., 2011; Lloret et al., 2018). Unlike the symplasmic pathway, which actively regulates the cell-to-cell transport, the transcellular pathway involves a passive transport between cells. It relies especially on aquaporins, plasma membrane intrinsic proteins (PIP) and tonoplast intrinsic proteins (TIP) that allow the circulation of water flow content and solutes depending on membrane transporter proteins availability (Yooyongwech et al., 2008; Wang et al., 2020). Genes coding for these proteins are inhibited during endodormancy in rice and could reduce the water transport (Afzal et al., 2016). Moreover, the apoplastic pathway is also regulated by changes in the cell wall structure allowing a restricted conductance of large macromolecule such as sugars until the transition to bud burst (Maurel et al., 2004; Signorelli et al., 2020).

Previous studies have brought some insights on how transport capacity is involved in tree dormancy but the dynamics and molecular actors involved in its regulation are still missing in fruit trees, which differ from forest trees in the environmental and physiological regulation of dormancy. In addition, most studies are lacking long-term dynamics on transport capacity throughout dormancy progression, that would allow a robust quantification of the communication within the bud itself and between twig and buds. In order to validate the hypothesis that transport capacity regulates dormancy progression in fruit trees, we combined physiological, imaging and transcriptomic approaches to characterize transport capacity during dormancy in sweet cherry flower buds.

## Materials and methods

### Plant material

Experiments were conducted on the sweet cherry cultivar ‘Fertard’ grafted on Maxma Delbard 14 rootstock, which is characterized by a very late flowering and high chilling requirements. The twigs and buds were sampled on ten sweet cherry cultivar ‘Fertard’ trees grown in an orchard at the Fruit Experimental Unit of INRAE in Toulenne, near Bordeaux (48° 51’ 46’’ N, 2° 17’ 15’’ E) under commonly used agronomical practices.

### Bud dormancy monitoring

To characterize bud dormancy depth and stage, we carried out forcing experiments, as previously described (Vimont et al., 2019), during six winter seasons between 2016 and 2022. In brief, every two weeks, three branches carrying flower buds were randomly cut from the trees and transferred to water-filled pots under forcing conditions in a climate chamber (25°C, long days 16 hours light/8 hours dark). After ten days under forcing conditions, the total number of flower buds and the number of flower buds that reached BBCH stage 53 corresponding to bud burst stage defined by scales separated and light green bud sections visible (Fadón et al., 2015) were recorded. We estimated the dormancy release date when 50% of the flower buds had reached the stage BBCH 53.

### Chill portion calculation

Chill accumulation during the different winter seasons was characterized using chill portions (Fishman et al., 1987) calculated with the chillR package (Luedeling et al., 2013) on hourly temperatures recorded on-site from September 1^st^ to flowering date.

### Transport capacity evaluation by imaging

Transport capacity to the flower bud tissues was evaluated based on the movement of a water-soluble fluorescent dye, calcein (Sigma C0875-5G). Branches carrying flower buds were sampled every two weeks between September and April from 2017 to 2022. Each branch was then cut into internodes carrying short twigs characterized by a vegetative bud surrounded by two to eight flower buds. The cut top was wrapped in parafilm to prevent dehydration and the basis of the short twigs was immersed in 6 mL of a 0.1 M calcein solution (0.1g of calcein powder diluted into 100mL of distilled water) in the darkness for 24h at room temperature. After incubation, the flower buds were cut in a median section and observed under a macroscope (Axiozoom Zeiss V.16). Images were acquired under white light and epifluorescence (**Supplementary Figure 1**). Calcein fluorescence (excitation 460-488 nm and emission 500-548 nm) was captured using a green fluorescence filter (548 nm) during 28 seconds. We also captured chlorophyll autofluorescence, as a control for the total bud surface, using a red fluorescence filter (593 nm) for 170 seconds.

Pictures were analyzed with the Image FIJI software (Schindelin et al., 2012). Every picture was reframed to remove the junction point to the branch in order to measure only the fluorescence within the bud. First, the numbers of red fluorescent pixels in the autofluorescence image were counted to measure the total bud area. Then the numbers of green fluorescent pixels in the calcein image were counted to measure the bud area irrigated by calcein tracer. (**Supplementary Figure 1**). A threshold of 40 was set for the intensity for all the pictures; this setting was the best compromise to cut off the background of low intensity pixels. The ratio between the green pixel numbers (i.e. calcein tracer area), and the red pixel numbers (i.e. chlorophyll/total bud surface) was calculated to quantify the transport capacity to the bud tissues.

### Callose imaging in flower buds

Flower buds were sampled from ‘Fertard’ trees on March 1^st^ 2017 before dormancy release (endodormancy phase), and on March 29^th^ 2017, after dormancy release (ecodormancy phase). 50 µm longitudinal sections of the buds were realized using a vibratome (Microm 650V, Thermo Scientific Microm) and immediately transferred to a fixation solution (4% paraformaldehyde in a phosphate buffer 0.1M pH 7.2) for one hour at room temperature. To visualize callose, sections were washed and observed with 25 µL of 10/1000 diluted aniline blue fluorochrome (Biosupplies, cat. No. 100-1, Australia. Sections stained with aniline blue were observed using an epifluorescence microscope (Zeiss Axiophot; excitation 450-490 nm and emission >520 nm) and a confocal microscope Zeiss LSM 880.

### RNA extraction and library preparation

Flower buds were sampled from October 2017 to March 2018 at 6 dates: October 17^th^, December 8^th^, January 3^rd^, January 15^th^, February 12^th^ and March 12^th^, with three biological replicates, corresponding to independent buds from three different trees. Buds were flash frozen in liquid nitrogen and stored at − 80°C prior to performing RNA extraction.

Total RNA was extracted from 100 mg of frozen and grinded flower buds using RNeasy Plant Mini kit (Qiagen) with minor modification: 1.5% PVP-40 was added in the extraction buffer RLT. RNA quality was evaluated using Tapestation 4200 (Agilent Genomics). Library preparation was performed on 1 μg of high-quality RNA (RNA integrity number equivalent superior or equivalent to 8.5) using the TruSeq Stranded mRNA Library Prep Kit High Throughput (Illumina cat. no. RS-122-2103). DNA quality from libraries was evaluated using Tapestation 4200. The libraries were sequenced on a HiSeq3000 (Illumina), at the sequencing facility Get-Plage (Castanet-Tolosan, France). Detail information on sequencing and mapping results including total number of reads and mapped reads per sample were summarized in **Supplementary Table S1**.

### Mapping and differential expression analysis

The raw reads obtained from the sequencing were analyzed using several publicly available softwares and in-house scripts. The quality of the reads was assessed using FastQC and possible adaptor contaminations and low quality trailing sequences were removed using Trimmomatic 0.36 (Bolger et al., 2014) with following settings fa:2:10:5:1 LEADING:3 TRAILING:3 SLIDINGWINDOW:4:15 MINLEN:36. Trimmed reads were mapped to the sweet cherry ‘Regina’ reference genome v.1 as previously described (Vimont et al., 2021) using STAR (Dobin et al., 2013).

The genome of the ‘Regina’ sweet cherry cultivar was assembled *de novo* resulting in a genome of 279 Mb (83% of estimated genome size) constituted of 92 scaffolds, with a high contiguity (Contig N50 = 1.23Mb, scaffold N50 = 5.96Mb) and a good completeness (95.9% BUSCO score for the genome sequence, scaffolds + unscaffolded contigs + haplotigs) (Le Dantec et al., 2019; Le Dantec et al., 2020).

Raw read count was performed using HTSeq count (Anders et al., 2015) and TPM (Transcripts Per Million) numbers were calculated and normalized with an in-house R script (**Datasheet 1**).

We performed a differential expression analysis on raw read counts to identify expression patterns that changed during dormancy. First, data were filtered by removing lowly expressed genes (average read count< 3) and genes not expressed in most samples (read counts = 0 in more than 75% of the samples). Then, differentially expressed genes (DEGs) between each time point (**Datasheet 2**) were assessed using DEseq2 R Bioconductor package (Love et al., 2014), in the statistical software R (R Core Team 2022). Genes with an adjusted p-value (padj, calculated using the Benjamini-Hochberg multiple testing correction method)< 0.05 and a log fold change > 1 between at least two dates were assigned as DEGs. In addition, we performed qRT-PCR on 7 marker genes to validate gene expression profiles obtained by RNA-seq for an independent study (**Supplementary Figure 2**).

### Principal component analysis and hierarchical clustering

Distances between the DEGs expression patterns over the time course were calculated based on Pearson’s correlation on TPM values. We applied a hierarchical clustering analysis on the distance matrix to define five clusters (**Supplementary Table S2**). For expression patterns representation, we normalized the data using z-score for each gene:


$$z\;score=\frac{\left(TPM_{ij}-mean_{i}\right)}{Standard\;Deviation}$$

where TPM_ij_ is the TPM value of the gene *i* in the sample *j*, mean_i_ and standard deviation_i_ are the mean and standard deviation of the TPM values for the gene *i* over all samples.

Principal component analyses (PCA) were performed on TPM values from different datasets using the *prcomp* function from R.

### Gene ontology enrichment analysis

The sweet cherry ‘Regina’ reference genome was annotated using Blast2GO (Gotz et al., 2008) for gene ontology (GO) terms (**Supplementary Table S3**). Using the topGO package for R (Alexa and Rahnenfuhrer, 2023), we performed an enrichment analysis on GO terms for biological processes in the DEGs compared to the whole set of annotated genes, based on a classic Fisher algorithm. Enriched GO terms were filtered with a p-value< 0.005 and the twenty GO terms with the lowest p-value were selected for representation.

### Selection of the candidate genes involved in transport capacity

In order to study the molecular actors potentially implicated in transport capacity, a list of candidate genes was built taking into consideration the full annotation of the sweet cherry ‘Regina’ genome and the corresponding descriptions found with TAIR, Mercator and Eggnog databases (Lohse et al., 2014; Huerta-Cepas et al., 2019). Among the 48, 133 genes from the sweet cherry ‘Regina’ genome, we selected 588 genes from literature (Robert and Friml, 2009; Rinne et al., 2011; Růžička et al., 2015; Afzal et al., 2016; Savage and Chuine, 2021) or by searching keywords associated with 1) symplasmic or transcellular pathway (glucan hydrolase, endoglucanase, beta glucosidase, plasmodesmata, callose synthase, plasma membrane intrinsic, tonoplastic intrinsic), 2) apoplastic pathway (cellulose synthase, pectinesterase, cell wall invertase) and 3) vascular pathway (xylem and phloem development).

## Results

### Transport capacity is reduced during dormancy

**Figure 1:**
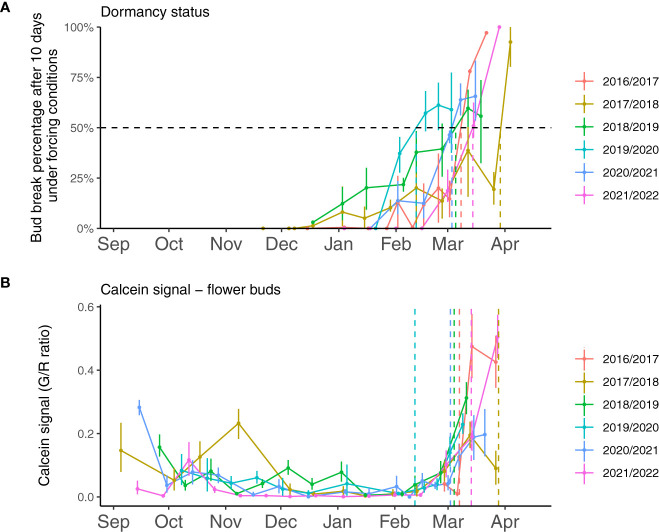
Experimental evaluation of sweet cherry flower bud dormancy status and transport capacity from 2017 to 2022. **(A)** Dormancy stage is monitored based on the budbreak percentage after ten days under forcing conditions (25°C, 16h light, and 8h dark). The dormancy release date (dotted lines) is estimated when 50% of the buds break after ten days in forcing conditions. Dormancy release dates were March 7^th^ 2017, March 29^th^ 2018, March 5^th^ 2019, February 11^th^ 2020, March 3^rd^ 2021 and March 14^th^ 2022. **(B)** The calcein tracer recorded in 15 flower buds for each date, estimated as the ratio between green and red fluorescence, was used to evaluate transport capacity. Dotted lines represent the estimated resumption of transport capacity.

To better characterize the dynamics of bud dormancy and transport capacity, we compared dormancy status and transport capacity in sweet cherry buds throughout dormancy stages from the end of summer to budbreak in the spring between 2017 and 2022 in the sweet cherry cultivar ‘Fertard’, characterized by high chilling requirements and late flowering. For dormancy status, we monitored the buds’ capacity to respond to forcing conditions (**Figure 1A**). Results showed a wide range of dormancy release dates over the five years, the earliest being February 12^th^ in 2020 and the latest being March 28^th^ in 2018 indicating that dormancy progression displays different dynamics between years. These dates were also much contrasted when calculated with chill accumulation (**Supplementary Figure 3A**), thus suggesting that the wide range of response was not solely due to differences in winter cold temperatures between years.

**Figure 2:**
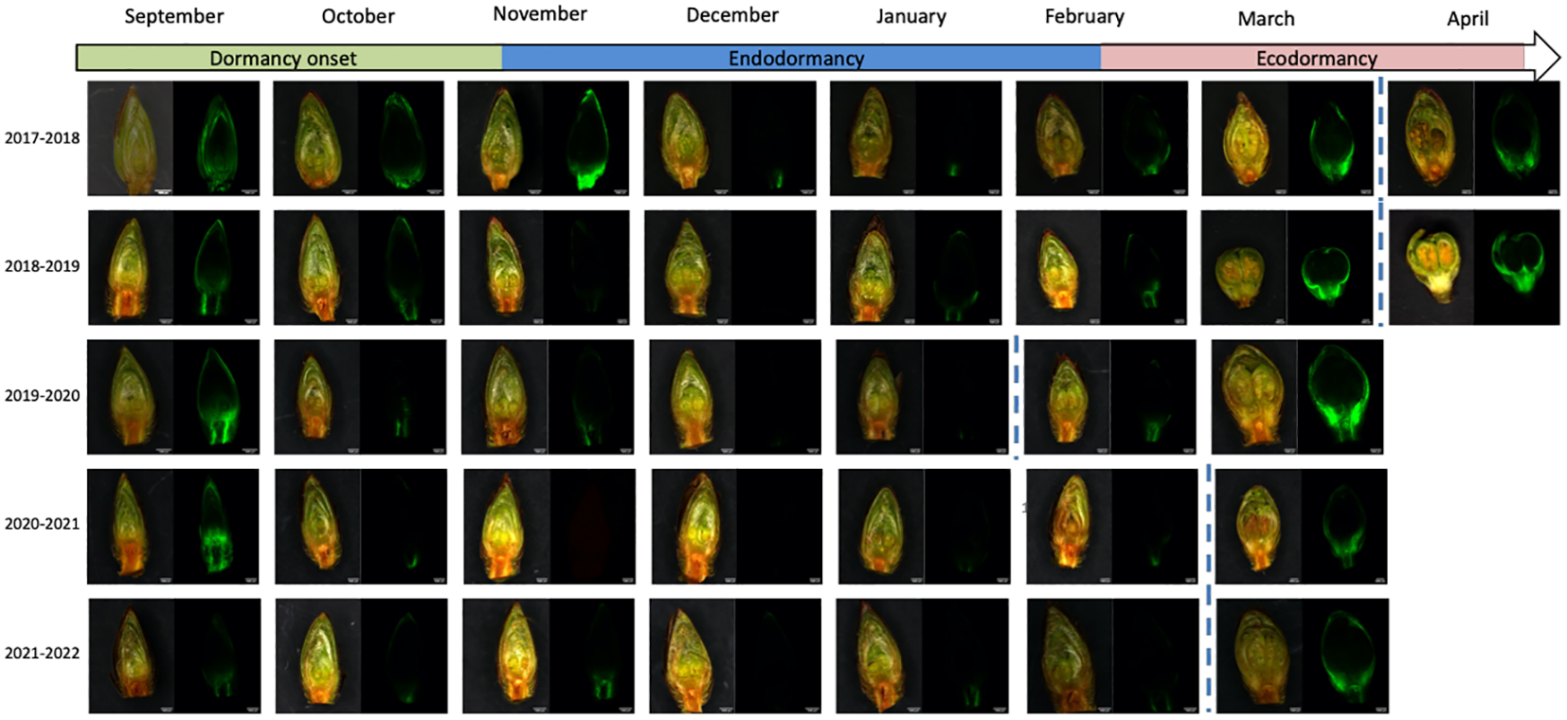
Calcein tracer visualization and flower bud development throughout dormancy for the sweet cherry ‘Fertard’ cultivar. The observations were done on longitudinal cross-sections of flower buds. For each date, buds were captured with the bright light channel (left picture) and the green channel (right) where calcein fluorescence can be visualized. Dormancy release date was determined by forcing experiments and is indicated by the dotted line.

To investigate whether dormancy release was linked to transport capacity, we also followed the movement of the water-soluble fluorescent molecule calcein from the twig to the dormant buds which allowed visualizing and quantifying the connectivity between the twig and the bud (**Figure 2**). Observations showed that the calcein tracer reaching the bud tended to decrease between September and November, except in 2017-2018, in line with previous reports that transport capacity may be impaired at the beginning of dormancy (Tylewicz et al., 2018) so we defined this period as dormancy onset. The endodormancy phase from November to January/February was characterized by the absence of fluorescence in the bud suggesting that the flower bud was partially isolated from the twig. After endodormancy was released, around February, the fluorescence signal was visible in the bud and increased during ecodormancy, as the buds were visibly growing and the floral organs were developing (**Figure 2**). For further investigation, we developed a method to quantify the calcein movement to the bud, which allowed comparing transport capacity dynamics between the seasons (**Figure 1B**). Quantitative results confirmed that calcein movement to the bud decreased in September or beginning of October, potentially corresponding to dormancy onset (**Figure 1B**). Interestingly, in 2017, the calcein tracer first increased in October before decreasing in November. For all other observation seasons, the calcein movement to the buds was low from November to February, corresponding to the endodormancy phase (**Figure 1B**). Subsequently, a sharp increase in the calcein transport to the bud was observed in February that corresponded to the transition toward ecodormancy and then budbreak (**Figure 1B**). Dynamics for transport capacity resumption as measured by calcein movement to the bud were very similar for the five seasons. On the contrary, the decreasing fluorescence signal during dormancy onset varied depending on the year. Overall, comparison between forcing data (**Figure 1A**) and calcein movement monitoring (**Figure 1B**) showed that, except for 2019-2020, the dynamics for transport capacity and dormancy release were not synchronized.

### Calcein transport to the bud may be regulated by environmental signals

**Figure 3:**
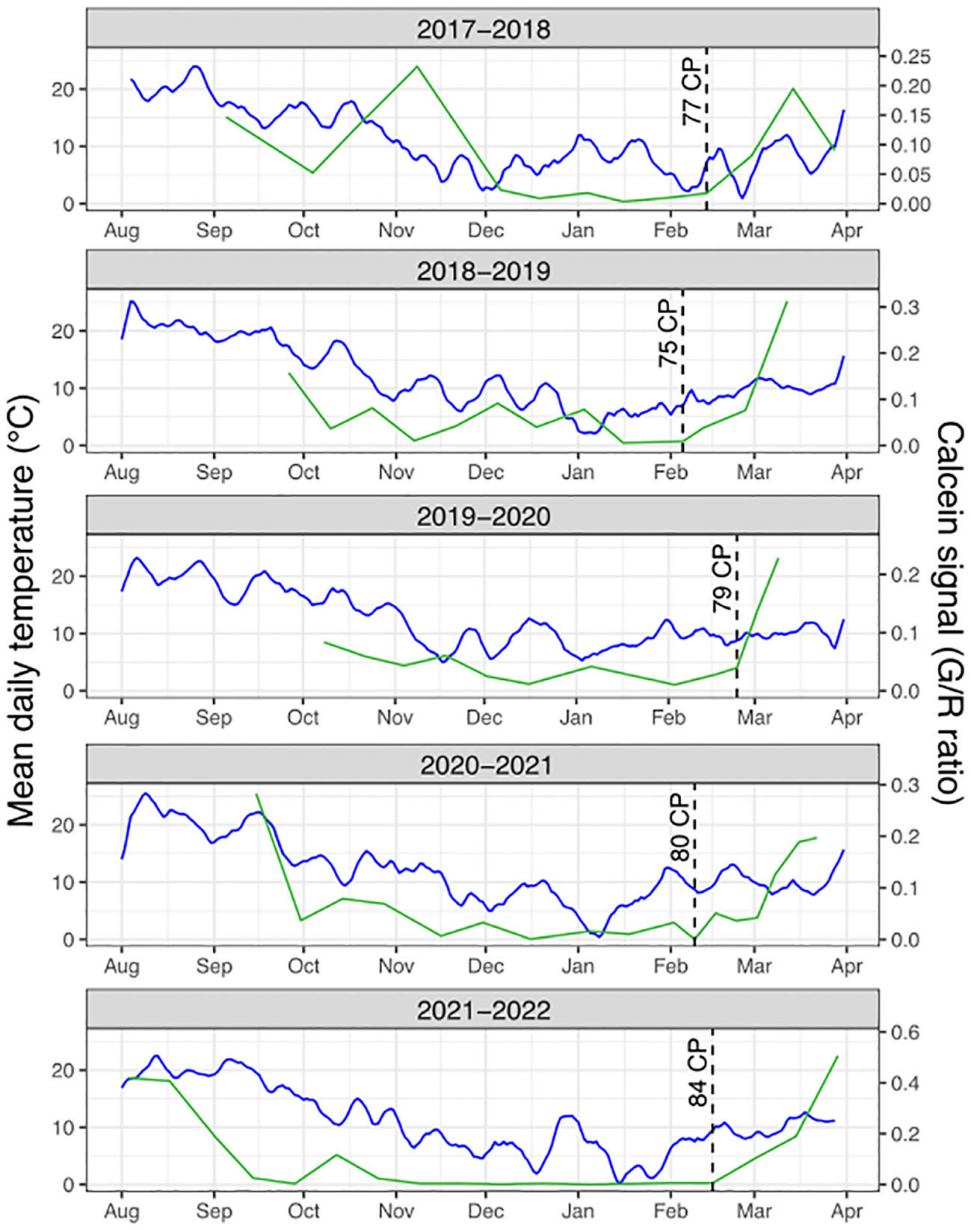
Calcein tracer dynamics and temperature conditions. For each sampling season, the average daily temperature (blue line) is represented along with the calcein tracer (Green to Red fluorescence ratio, green line). The cold accumulation in chill portions (CP) at the estimated resumption of transport capacity is represented by the dotted lines.

Following our observations that calcein movement to the bud was not fully associated with dormancy status (**Figure 1**), we investigated whether the resumption of calcein movement to the bud in February/March could rather be linked to changes in environmental conditions. Calcein tracer profiles over the years show a recurring pattern with a clear resumption occurring every season between and February 5^th^ and 24^th^. This regular timing between years regardless of the chill accumulation could suggest that photoperiod may trigger the resumption of calcein transport to the bud. Day length at these dates for the sampling site ranges from 10 to 11 hours. Nevertheless, a previous study (Heide, 2008) has shown that dormancy in *Prunus* in general and sweet cherry in particular was more driven by temperature than photoperiod. Therefore, we also investigated temperatures during that period. In particular, we considered the chill accumulation for the different seasons and we found that the calcein movement to the bud resumed within a range of 75 to 84 chill portions accumulated (**Supplementary Figure 3B**). This result suggested that a certain amount of chill accumulation may be necessary to reactivate calcein transport to the bud. Furthermore, we hypothesized that variations in the timing of transport capacity resumption between years may subsequently depend on when warmer temperatures occur, which are essential for cellular activity and flower bud growth resumption. Indeed, bud development seems to resume while the calcein tracer increases and leads to the formation of flower’s verticilles when temperatures are getting warmer and heat accumulation occurs (**Figures 2**, **3**). Hence, calcein tracer resumption seems to be regulated by a fine and complex balance between a threshold for cold accumulation (between 75 and 84 CP) and heat accumulation due to warmer temperatures.

### Callose deposition in vascular tissues decreases after dormancy release

**Figure 4:**
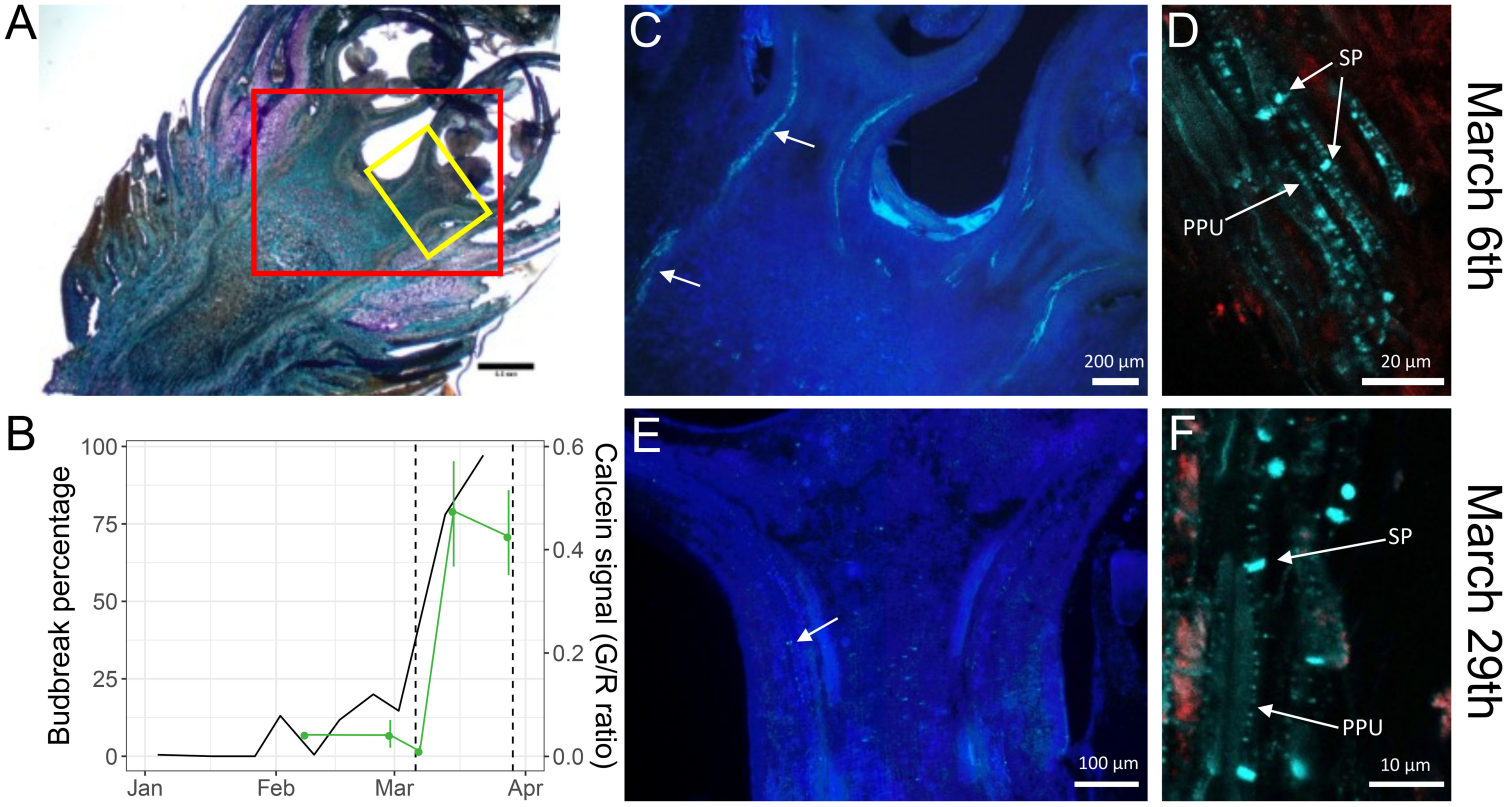
Observations of callose accumulation in sweet cherry flower buds. **(A)** 50 µm section of a flower bud from the late flowering cultivar ‘Fertard’, stained using toluidine blue, with two squares representing the approximate areas corresponding to the March 6^th^ imaging (red square) and March 29^th^ (yellow square). **(B)** bud break percentage after ten days under forcing conditions (black) and calcein tracer (green) during the 2016-2017 sampling campaign. The dash vertical lines represent the two sampling dates for imaging. Flower buds were cut into 50 µm sections and callose was observed with aniline blue fluorochrome on **(C)**, **(D)** March 6^th^ and **(E)**, **(F)** March 29^th^. Arrows show aniline blue fluorescence in the vascular systems. SP: sieve plate; PPU: Pore-plasmodesmata unit.

As transport capacity during bud dormancy was previously shown to be mostly regulated by the dynamic deposition and degradation of callose at the plasmodesmata (Aloni et al., 1991; Aloni et, 1997; Rinne et al., 2001; Rinne et al., 2011; Veerabagu et al., 2023) we investigated the presence of callose in flower buds of the ‘Fertard’ cultivar before and after dormancy release, also corresponding to low and high transport capacity as evaluated by the calcein tracer (**Figure 4B**). We observed high fluorescence in the vascular system of buds sampled on March 6^th^, when the calcein tracer in the bud and the budbreak percentage were low (**Figures 4A–C**; **Supplementary Figure 4**). In particular, the callose was present at a higher level in both sieve plates pores that connect phloem sieve elements end-to-end and pore-plasmodesmata units (PPUs) between companion cells and sieve elements(**Figure 4D**). In contrast, fluorescence associated with callose was much reduced on the buds sampled on March 29^th^, corresponding to high budbreak percentage and calcein transport (**Figures 4B, E**; **Supplementary Figure 4**), with a few spots visible in vascular areas at the base of the flower primordium. Although callose was still observed around sieve plates, the fluorescence was very weak at the PPUs (**Figure 4F**; **Supplementary Figure 4**). These results seemed to confirm that callose degradation in the buds was correlated with dormancy release and transport capacity resumption.

### Molecular characterization of bud dormancy suggest transport implication in dormancy progression

In order to identify molecular actors involved in dormancy progression, we realized a whole transcriptome RNA-seq analysis on flower buds from the sweet cherry cultivar ‘Fertard’ sampled at six dormancy dates during dormancy between October 2017 and March 2018 (**Supplementary Figure 5**). We performed a differential expression analysis between the sampling dates including all annotated genes (48,133) of the reference sweet cherry ‘Regina’ genome using DESeq2 with a threshold of 0.05 on the adjusted *p-value.* 10,250 differentially expressed genes were identified (**Supplementary Table S2**). When projected into a two-dimensional space (Principal Component Analysis, PCA), data for these DEGs showed that the transcript patterns of each sample clearly separated the dormancy stages (**Supplementary Figure 6A**) thus suggesting that specific genes and signaling pathways were activated throughout dormancy progression. The first dimension of the analysis (PC1) explained 40% of the variance and clearly represents the different types of dormancy from ecodormancy to endodormancy, while the second dimension (PC2) explained 23.83% of the variance visibly associated with dormancy depth.

**Figure 5:**
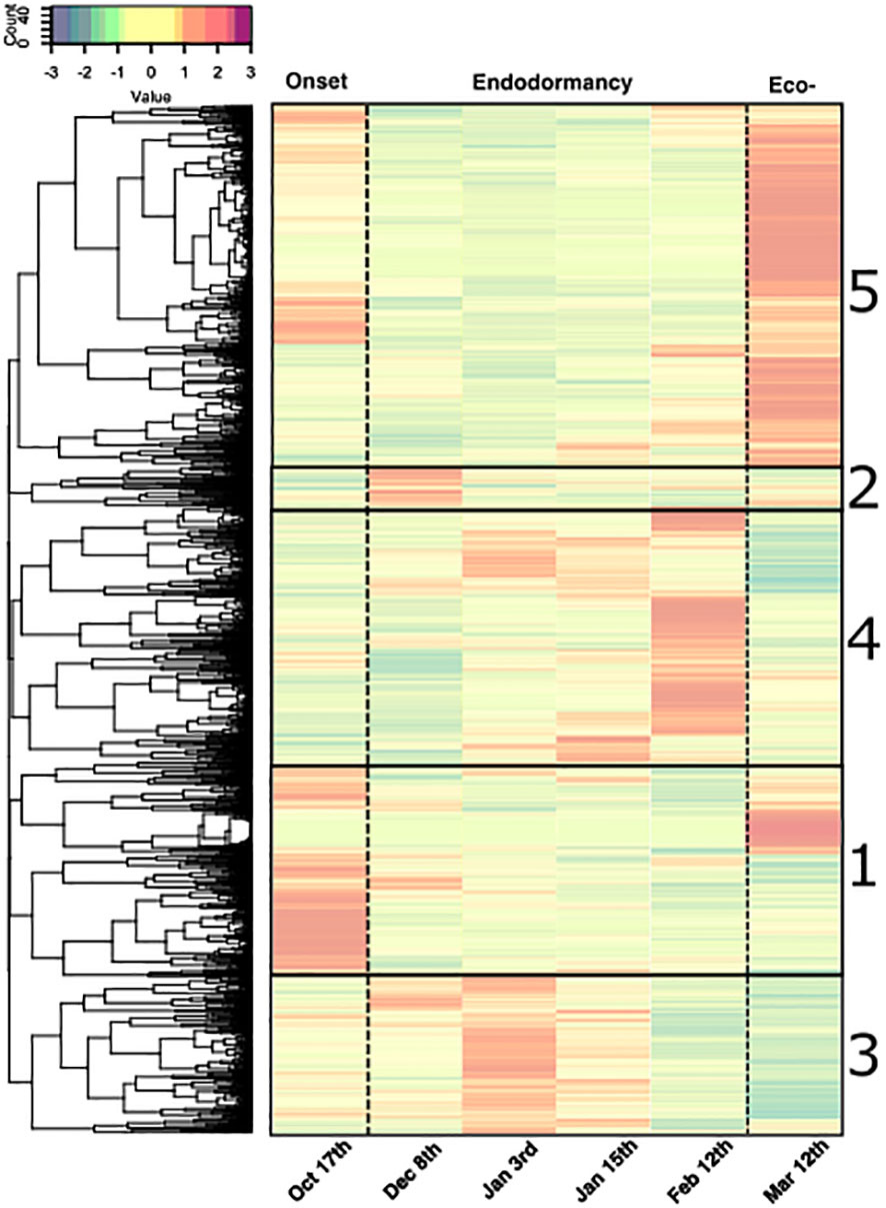
Clusters of expression patterns for differentially expressed genes during dormancy in flower buds of the sweet cherry cultivar ‘Fertard’. Heatmap representing the average z-score calculated for each gene from three trees at a given date. Each row corresponds to the expression pattern across samples for one gene. Upper panel represents the colors corresponding to the z-score values. Gene clusters are ordered based on the chronology of the expression peak (from earliest 1) dormancy onset, 2) early endodormancy, 3) mid endodormancy, 4) late endodormancy and 5) ecodormancy). Expression values were normalized and *z-scores* are represented here.

We further investigated the genes specifically activated or inhibited during the phases of dormancy using a hierarchical clustering approach based on five clusters depending on the gene expression data (**Figure 5**; **Supplementary Table S2**). First, clusters 1 (1959 genes) and 5 (3428 genes) included genes that were down-regulated during endodormancy and were up-regulated during dormancy onset or activated during ecodormancy for clusters 1 and cluster 5, respectively. The second group comprised clusters 2 (413 genes), 3 (1736 genes) and 4 (2714 genes) where genes were specifically expressed during early, mid and late endodormancy respectively.

To go a step forward and explore the function of genes that were differentially expressed during dormancy, we performed a GO enrichment analysis to pinpoint molecular functions and biological processes over-represented in DEGs compared to the entire gene set of reference genome (**Supplementary Table S4**). Results showed that GO terms associated with oxidation-reduction processes, transcription factor activity, carbohydrate metabolic processes and response to abiotic stimulus were significantly enriched in the DEGs during bud dormancy (**Supplementary Table S4**). Interestingly, we also found that terms indirectly related to transport capacity were significantly highlighted, such as glucan endo-1, 3-beta-D-glucosidase activity for molecular function and transmembrane transportfor biological process (**Supplementary Table S4**).

### Identification of genes involved in transport capacity during dormancy

In accordance with calcein tracer profiles, callose deposition observations and GO enrichment results, we further investigated whether genes involved in the regulation of transport capacity showed particular expression patterns throughout dormancy progression. We focused on a list of 588 candidate genes potentially involved in the regulation transport, in a direct or indirect manner (**Supplementary Table S5**): i) genes associated with the symplasmic pathway related to the plasmodesmata structure and functioning, callose metabolism and a sub class in symplasmic pathway: the transcellular pathway related to aquaporins or channels involved in cell-to-cell transport by crossing both the apoplast and the symplast; ii) genes related to the apoplastic pathway, involved in cell wall formation and degradation which participate to the transport capacity in an indirect manner by regulating the interspace between cells; and iii) genes implicated in the vascular pathway, especially in xylem formation which participate to the transport capacity indirectly by regulating the proliferation of procambial and cambial cells to establish functional vascular system.

We identified 152 differentially expressed genes (DEGs) between at least two dates. The three classes of the different transport pathways were represented among the DEGs (**Supplementary Table S6**). Very similarly to the previous PCA results on all DEGs, a projection of the expression data for transport candidate DEGs showed that the flower bud stages were well separated in the projection for the first two PCA dimensions (**Supplementary Figure 6B**), indicating that the genes involved in transport capacity displayed expression patterns specific to the different bud dormancy stages. Interestingly, genes involved in transport capacity were classed the five main clusters previously characterized (**Supplementary Table S6**): genes expressed during dormancy onset (clusters 1; 27 genes), genes specifically expressed during early, mid and late endodormancy (respectively clusters 2, 3 and 4; 58 genes) and genes highly expressed during ecodormancy (cluster 5; 67 genes).

### Genes involved in transport capacity genes are mainly repressed during endodormancy

**Figure 6:**
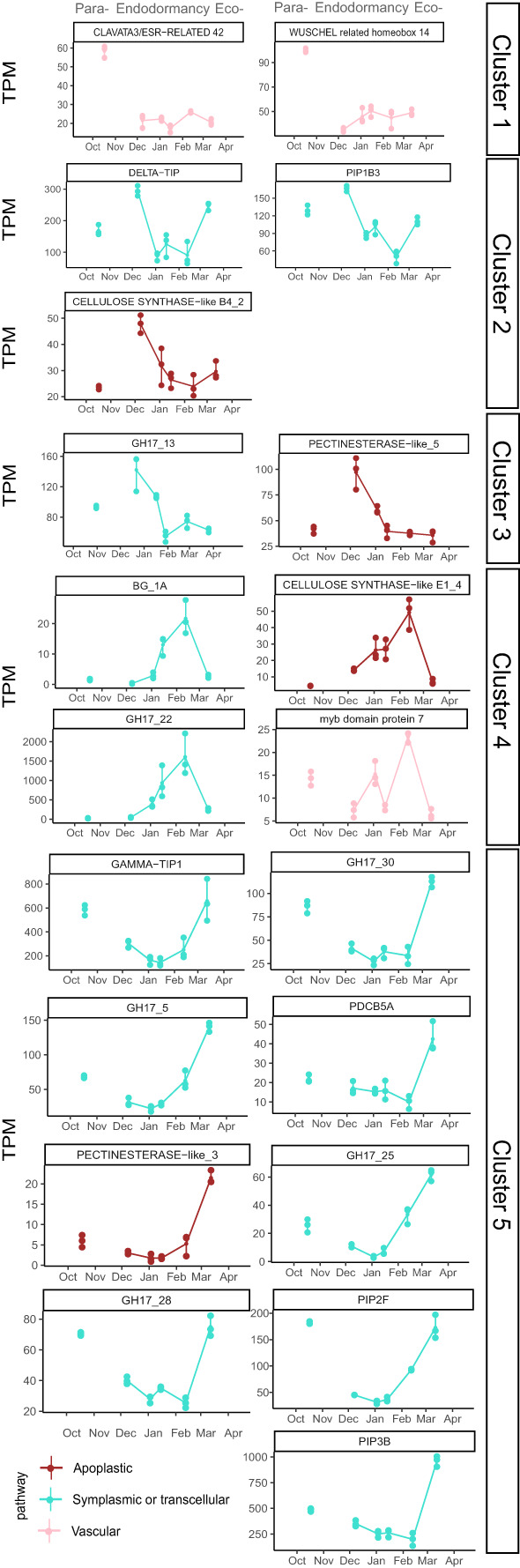
Expression profiles of key genes involved in transport capacity. Cluster 1 and 5 genes are down regulated during endodormancy, contrary to cluster 2, 3 and 4 which are upregulated. Genes have been chosen based on their expression profile and their functional annotation. Points represent the average TPM and error bars correspond to the data range (n=3 for each date).

Among the 152 DEGs, 94 genes belonged to clusters 1 and 5, corresponding to genes highly expressed during dormancy onset, after dormancy release and during ecodormancy, and therefore fully repressed in December during endodormancy (**Figure 5**). In these clusters, genes involved in the symplasmic pathway are well represented including *GH17* or Glucan Hydrolase related genes that regulate callose degradation, and therefore could be involved in the reopening of the cell-to-cell connection through the plasmodesmata. For example, *GH17_5*, *GH17_25*, *GH17_28*, *GH17_30* displayed a typical expression profile of repressed genes that could explain the transport capacity reduction during endodormancy (**Figure 6**). *PlASMODESMATA CALLOSE BINDING 5A* (*PDCB5A*), a callose-binding gene, and *PLASMODESMATA LOCATED PROTEIN 8* (*PDLP8*), involved in plasmodesmata trafficking, also showed a similar expression pattern thus suggesting a lower transport activity through plasmodesmata during endodormancy. In the same way, *PDCB5A* and *PDCB5B* that regulate cell-to-cell communication (Simpson et al., 2009) were characterized by a low expression during endodormancy and a higher expression at the ecodormancy stage (**Supplementary Figure 7**). We found genes associated with water transport such as gene *PIP3B*, and *GAMMA-TIP1* that are downregulated during endodormancy (**Figure 6**). These genes are characterized as “water channels” and their gene expression profiles could reinforce the hypothesis that the water flow is hindered during endodormancy and resumed at the ecodormancy stage.

In addition to a majority of genes belonging to symplasmic or transcellular pathway that were down-regulated during endodormancy, we also identified *WUSCHEL 14, CLAVATA3/ESR-RELATED 42* genes classed in cluster 1. These genes may be involved in the proliferation of procambial and cambial cells and could control xylem formation, and therefore participating in regulating the vascular transport between the twig and the flower bud during dormancy onset (Růžička et al., 2015). Moreover, a transcription factor MYB85 implicated in xylem maturation and regulating lignin biosynthesis could participate in the control of vascular transport as its expression is repressed during endodormancy. Finally, genes involved in the cell wall formation are also represented with *PECTINESTERASE-like_3* gene and several *CELLULOSE SYNTHASE* genes which could reduce intercellular spaces and regulate apoplastic transport.

### Transport capacity genes activated during endodormancy

The genes expressed during endodormancy can be divided into 3 clusters corresponding to a peak of expression during early (cluster 2), mid (cluster 3) and late (cluster 4) endodormancy (**Figure 6**). In cluster 2, two aquaporins genes *DELTA-TIP* and *PIP1B3* are highly expressed during early endodormancy which contrasts with the down regulation of other aquaporins during endodormancy and could participate to the necessary balance of transcellular pathway capacity. In addition, genes involved in cell wall formation and development such as *PECTINESTERASE-like_5* gene, *CELLULOSE SYNTHASE-like B4_2 and CELLULOSE SYNTHASE-like E1_4* genes showed high expression levels during mid and late endodormancy, potentially associated with an increase thickness of the cell wall which could reduce apoplastic transport capacity during endodormancy (Hatfield et al., 2017) (**Figure 6**; **Supplementary Figure 8**). Interestingly, in cluster 4, *MYB7*, a negative regulator of xylem formation associated with vascular connection establishment (Růžička et al., 2015) was highly expressed at the end of endodormancy, which could be linked to reduced vascular transport. Finally, contrarily to most of *GH17* genes, *GH17_22* displayed a high expression during late endodormancy as well as *β GLUCANASES BG_1A* which are not part of the *GH17* family according to annotation databases, indicating a potential initiation of callose degradation at the end of dormancy (**Figure 6**; **Supplementary Figure 8**).

### Identification of genes potentially associated with calcein transport

**Figure 7:**
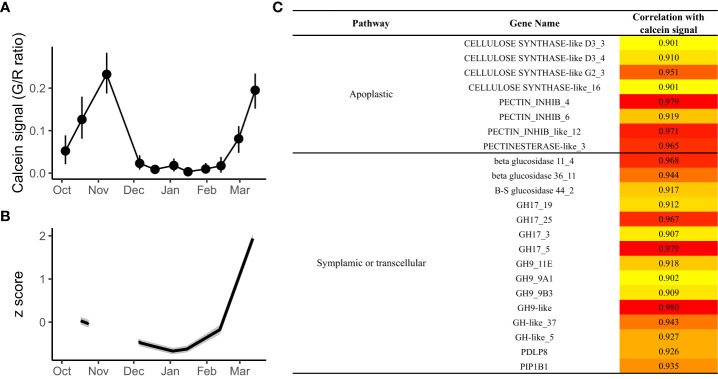
Genes showing the best correlation with the calcein tracer during flower bud dormancy. **(A)** The calcein tracer for 2017/2018 was evaluated using green to red fluorescence ratio on longitudinal bud sections. The line corresponds to the average of 15 buds. **(B)** Average expression profile for the genes with the expression levels, expressed as z scores, best correlated with the calcein tracer. **(C)** Details for the 21 genes with a correlation of at least 0.9 with the calcein tracer.

In order to highlight the implication of candidate genes in the transport capacity regulation during dormancy, correlations between transcriptomic data and physiological observations were performed. These correlations, calculated without the November time point due to the absence of RNA-seq data available for this point, led to the identification of several candidate genes with expression profiles highly correlated with calcein tracer profile (**Figures 7A, B**). In total, we identified 23 genes with a positive correlation over 0.9. These genes all belonged to cluster 5 except *PIP1B1* that belonged to cluster 1(**Figure 7C**), with the main expression pattern of inhibition during endodormancy and upregulation during dormancy onset and/or ecodormancy. Among these 23 genes, four *GH17* genes, six *GH* related genes, three *GLUCOSIDASE* genes, *PDLP8*, and *PIP1B1* are involved in symplasmic or transcellular pathway, while one *PECTINESTERASE* genes, three *PECTINESTERASE INHIBITOR* genes, and four *CELLULOSE SYNTHASE* genes were associated with the apoplast pathway which could regulate transport capacity by modifying cell wall and cell interspace.

## Discussion

### Calcein tracer could be a reliable marker for transport capacity during dormancy

Previous studies have attempted to visualize transport capacity to the bud with a main focus on the transition from endodormancy to ecodormancy (Ashworth and Rowse, 1982; Rinne et al., 2001; Xie et al., 2018; Signorelli et al., 2020). Their results led to the hypothesis that transport capacity resumption was associated with endodormancy release and the initiation of bud burst. Here in order to validate this hypothesis in temperate fruit trees, we quantified the movement of the fluorescent molecular calcein as a proxi for transport capacity in flower buds in sweet cherry throughout the dormancy period. In particular, the fluorescent signal allowed robust automatic quantification of the calcein presence at the bud, as opposed to non-fluorescent dyes such as acid fuchsin, eosin or Amido Black (Essiamah and Eschrich, 1986; Xie et al., 2018; Signorelli et al., 2020). Nevertheless, microscopy observations using ionic form of calcein dye remain descriptive and cannot provide any precise information on the cell-to-cell transport and on phloem loading or xylem trafficking but give a reliable state of connectivity between bud and twig. Therefore, we demonstrated that the calcein tracer measured in buds showed a recurring pattern consisting in a decrease at dormancy onset followed by an absence of signal during endodormancy and a marked increase synchronized with bud development and growth. Moreover, this specific profile seemed to be reproducible over the years, suggesting that the calcein tracer may be used as a marker of transport capacity and its dynamics was reliable information to understand and characterize the connections that occur between the flower buds and the twig during the wintering period. This systematic approach could be applied to specific dyes to precisely quantify the dynamics for the different transport pathways during bud dormancy stages.

### Multiple biological pathways are involved in the regulation of dormancy progression

We showed that genes were specifically expressed during certain dormancy stages, as distinguished by the PCA and the hierarchical clustering analysis. Most of DEGs were down-regulated during endodormancy, suggesting that they played an active role in paradormancy and ecodormancy, with a marked decrease in global cellular activity during endodormancy. However, some signaling pathways were still active during endodormancy, with genes identified in clusters 2, 3 and 4, which could be major actors of dormancy maintenance and progression.

Previous transcriptomic studies have reported multiple biological pathways potentially involved in dormancy progression such as carbohydrate metabolism, oxidative stress, phytohormones, epigenetic regulation and transport (Zhu et al., 2015; Zhang et al., 2018b; Vimont et al., 2019; Yu et al., 2020; Zhu et al., 2020). In line with these finding, our results have highlighted that genes associated with oxidation-reduction processes, carbohydrate metabolism and response to abiotic stresses were expressed during bud dormancy in sweet cherry. This strongly suggests that common pathways regulate dormancy onset, maintenance and progression in fruit trees, regardless of the species, the cultivar, the location or the observation season. In particular, our results highlighted GO terms associated with oxidoreductase activity which is implicated in oxidative stress regulation, considered as one of the key processes involved in the transition from endodormancy to ecodormancy (Ophir et al., 2009; Vergara et al., 2012; Beauvieux et al., 2018; Meitha et al., 2018). Terms associated with transcription factor activity were also significantly enriched in the genes differentially expressed throughout dormancy, which could be related to key regulators such as *DORMANCY-ASSOCIATED MADS-box* (*DAM*) genes involved in dormancy establishment and maintenance (Falavigna et al., 2019; Fadón et al., 2020)or transcription factors from the WRKY and MYB families, previously identified as serious candidate regulators in sweet cherry bud dormancy (Vimont et al., 2019). Finally, we found significant enrichment for terms associated with carbohydrate metabolic process which have been reported to vary through dormancy progression (Tixier et al., 2019), but also terms related to glucan endo-1,3-beta-D-glucosidase activity and glucan metabolic. Interestingly, these terms could potentially be related to transport capacity as these processes have been described to participate to callose degradation, thus allowing the restoration of cell-to-cell communication after chilling accumulation to promote dormancy release (Rinne et al., 2001; Rinne et al., 2011; Lloret et al., 2018).

### Transport capacity decrease may be associated with bud dormancy onset

We found that the calcein movement to the bud decreased within a short time span around late September/beginning of October, which we associate with the onset of dormancy. One exception was noticed for the season 2017-2018, when levels of calcein tracer in the bud showed a significant increase in October and November before decreasing in December. This marked reduction in transport pathways at the onset of endodormancy has been reported in different perennial species (for a review see Savage and Chuine, 2021). Interestingly, discrepancies between years might reveal the regulation by temperature of dormancy onset since milder temperatures were recorded during October 2017, which could be associated with a later dormancy onset as shown by the calcein levels.

Accordingly, we found molecular evidence that key genes involved in the symplasmic, apoplastic and vascular pathways were gradually down-regulated in sweet cherry flower buds during this dormancy onset period. Most of these genes were associated with symplasmic transport, such as *GH17_3*, *GH17_5*, *GH17_9*, *GH17_19* and *GH17_25*, suggesting a reduction of callose degradation, and therefore increased callose levels in the buds. These results are consistent with the reports that callose is deposited at plasmodesmata during autumnal growth cessation and dormancy onset, thus blocking inter-cellular communication and preventing the movement of growth-promoting signals and metabolic activities (Jian et al., 1997; Rinne and Schoot, 2003; Singh et al., 2017; Tylewicz et al., 2018). Interestingly, while there have been reports on the importance of a balance between callose production through *CALS* genes and degradation by *GH17* genes for dormancy establishment (Ruttink et al., 2007; Singh et al., 2018; Tylewicz et al., 2018; Singh et al., 2021), here none of the sweet cherry *CALS* genes were differentially expressed throughout dormancy, thus suggesting that callose turnover could be mostly regulated through degradation by the *GH17* genes. Moreover, we found that *PIP1B* genes, which regulate water transport through the transcellular pathway, were also down-regulated, leading to limited water transport participating to the transition toward endodormancy as previously observed with low aquaporin expression (Yooyongwech et al., 2008). In grapevine, Xie et al. (2018) found that the vascular connectivity between buds and cane was disrupted during endodormancy, potentially associated with the absence of functional xylem. Nevertheless, microscopy observations using ionic form of calcein dye remain descriptive and cannot provide any precise information on the cell-to-cell transport and on phloem loading or xylem flow but give a reliable state of connectivity between bud and twig. Consistently, the marked down-regulation during dormancy onset of two genes, *WUSCHEL 14, CLAVATA3/ESR-RELATED 42*, involved in the xylem formation (Růžička et al., 2015) may suggest that the vascular system is paused between the twig and the flower buds at the beginning of dormancy. All together, these molecular actors participate to reduce transport capacity and prepare the flower bud to acclimate to unfavorable conditions. However, the environmental regulation of the expression for these key genes is still unknown. Dormancy onset in perennial species can be triggered by cooler temperature, shorter photoperiod and endogenous control (Thomas and Vince-Prue, 1996; Battey, 2000). Contrarily to forest trees, where dormancy onset have been reported to be regulated by decreasing day length, *Prunus* species such as sweet cherry have been described to induce their dormancy onset and growth cessation mostly under the influence of low temperature (Heide, 2008; Cooke et al., 2012). Therefore, we might wonder whether the molecular pathways identified as key actors for the regulation of autumnal growth cessation and dormancy onset associated with transport capacity in forest trees, such as ABA and ABA/GA balance (Tylewicz et al., 2018; Veerabagu et al., 2023) may also be involved in the control of dormancy onset in fruit trees.

### Transport capacity is highly reduced during endodormancy

Calcein tracer profiles revealed that transport capacity is reduced, if not absent, during endodormancy, as it has been widely reported in other species (Ashworth and Rowse, 1982; Rinne et al., 2011; Xie et al., 2018; Fadón et al., 2020; Savage and Chuine, 2021). At a molecular level, we found that most of the candidate genes exhibited low expression levels during endodormancy (clusters 1 and 5) including *GH17*, *PIPs*, *PDLP* involved in symplasmic or transcellular pathways, *CELLULOSE SYNTHASE* and *PECTINESTERASE* involved indirectly in apoplastic pathway by regulating the intercellular space, as well as genes involved in xylem formation and development such as *WUSCHEL 14* and *CLAVATA3/ESR-RELATED 42* which could participate to the establishment of the vascular system. These results suggest that the arrest of transport and communication may be due to the total or partial shutdown of all transport pathways. *CELLULOSE SYNTHASE* and *PECTINESTERASE* may act indirectly as transport capacity regulators but could also promote bud break by playing a role in growth resumption due to their function in cell division and cell expansion during ecodormancy (Bosch et al., 2005; Endler and Persson, 2011).

In particular, the symplasmic pathway could be reduced due to the presence of callose deposition in all cells including sieve plate and sieve pores of phloem cells as we observed in endodormant buds, which is also consistent with the low expression of most of *GH17* genes. This communication blockage might be associated with reduced metabolic and physiological activities in cells during endodormancy (Rinne and Schoot, 2003; Wang et al., 2022). Our results also support the findings that water transport can be reduced during endodormancy (Yooyongwech et al., 2008) as we found that most of the *PIP* and *TIP* genes were repressed at this stage in flower buds. These gene families encoding water channels such as aquaporins have been reported to have potential function in the control of free water content during dormancy in Japanese pear (Saito et al., 2015) and in response to cold temperature in *Arabidopsis Thaliana* (Lee et al., 2012). Moreover, reduction of water transport during endodormancy may be crucial for enhanced cold tolerance (Sreedharan et al., 2013; Afzal et al., 2016; Xu et al., 2021), to protect from frost events and stop ice propagation in bud cells (Quamme, 1978). Finally, we found that most of the *PECTINESTERASE* and *CELLULOSE SYNTHASE* genes were down-regulated during endodormancy (clusters 1 and 5). These genes have been described to play a role in cell wall modification under cold stress and could participate indirectly in the apoplastic transport regulation during dormancy and these expression profiles are consistent with previous studies of cold treatment in poplar and cotton (Solecka et al., 2008; Ko et al., 2011; Zhu et al., 2013; Willick et al., 2018). Interestingly, eight additional *CELLULOSE SYNTHASE* and three *PECTINESTERASE* genes were also found to be highly expressed during endodormancy, which suggests that cellulose deposition in cell wall might be regulated by a complex balance.

Likewise, only a small number of candidate genes were expressed during endodormancy. These included four *GH17* genes, and the *BG* genes, another class of genes potentially involved in plasmodesmata aperture, annotated as β-1,3 glucanases but not as part of the *GH17* family, with upregulated expression during mid and late endodormancy (clusters 3 and 4) when the transport capacity was very low. These results suggest that some dynamics in the regulation of callose deposition were still needed during this period. In addition, *PIP1B3* and *DELTA-TIP* have an expression peak during endodormancy contrarily to the other aquaporins genes. This result could be explained by a role in cold acclimation for these *PIPs* and *TIPs* genes as it has been reported in Arabidopsis (Ruttink et al., 2007; Rahman et al., 2020).

### Transport capacity resumption is associated with bud growth rather than dormancy release

Our results showed that transport capacity resumption was not synchronized with dormancy release dates, but was more related to bud growth and development. Despite a quite large range of dormancy release dates (February 12^th^ to March 28^th^) over the five years of observation, we found that transport capacity resumption, quantified using calcein movement, occurred in a shorter period (February 5^th^-24^th^). In the debate on how and when transport capacity (between cells and between buds and twigs) is resumed, our results support the hypothesis that the different transport pathways are reactivated after dormancy release or after some chill accumulation, and when milder temperatures trigger the transition towards flowering in early spring.

Transport capacity within the bud is essential at the beginning of growth resumption for metabolism but as long as the metabolic and physiological activities are limited to the organ itself, the symplasmic and apoplastic pathways might be sufficient to support the local processes (Vitasse et al., 2014; Xie et al., 2018). Accordingly, we found that genes associated with the symplasmic pathway were among the first activated at the end of endodormancy, including most of the *GH17* genes. These results are consistent with the hypothesis that callose degradation leading to a reopening of plasmodesmata is crucial for bud burst (Rinne et al., 2011). This hypothesis is further supported by the expression pattern for *PDLP8*, another gene involved in symplasmic pathway and belonging to a gene family that has been reported to regulate trafficking through plasmodesmata (Thomas et al., 2008).

As metabolic activities substantially increase in the bud, the vascular system is reactivated via differentiation of the xylem conduits and callose degradation in the phloem as reported in *Prunus* species (Ashworth, 1984; Andreini et al., 2012; Viti et al., n.d), associated with higher needs in water and nutrients. These processes could therefore explain the increase in calcein movement that we observed in sweet cherry between the branch and the flower buds. Consistently, we identified genes highly correlated with the calcein tracer reflecting their regulatory role in the reconnection between bud and branch. These include five *GH17* genes, suggesting that callose may be actively degraded to facilitate the phloem connection toward the flower bud, which was further supported by our observations that callose was less present at sieve elements in the phloem before bud burst. Moreover, the *MYB85* gene expression is also correlated with the calcein tracer and could participate to the establishment of vascular elements as it has been identified as a potential regulator of xylem maturation and lignin biosynthesis (Růžička et al., 2015).

### Regulation of transport capacity in sweet cherry dormant buds

Our results suggest that the reactivation of transport capacity involved in bud growth and development could be separated into two periods before budburst, potentially associated with differential temperature regulation. First, at the end of endodormancy and as mentioned above, cellular processes are limited to the organ itself, facilitated by the reactivation of symplasmic and apoplastic pathways. They were potentially resumed after a photoperiod threshold or some chilling accumulation as supported by the cold accumulation threshold between 75 and 84 chilling portions observed for the five years of data. Indeed, before this threshold, transport capacity is never resumed even when mild temperatures could occur in December or January. Second, as temperatures increase and development accelerates, the sink increases substantially at the bud and functional vascular and nonvascular pathways are needed to allow for exchange between the organs (Savage and Chuine, 2021). Based on our observations and the related temperature data, reactivation of this long-distance transport may require warmer temperatures and consequently heat accumulation (Ashworth, 1984; Welling and Palva, 2006).

Therefore, similarly to the regulation of dormancy, a fine and complex balance of cold and heat accumulation appear to control transport capacity before bud burst. How these potential chilling and heat requirements for transport capacity are regulated and how they are related with dormancy progression need to be further investigated (Horvath et al., 2003; Campoy et al., 2011).

## Data availability statement

The datasets presented in this study can be found in online repositories. The names of the repository/repositories and accession number(s) can be found below: https://www.ncbi.nlm.nih.gov/geo/, GSE229429.

## Author contributions

BW designed the study. MF, HB, and DB performed the experiments. MF and BW analyzed the data and wrote the manuscript. All authors contributed to the article and approved the submitted version.

## Supplementary material

Supplementary Figure 1 Microscopy observations of flower bud under a) bright light, b) red filter corresponding to chlorophyll excitation in order to obtain the total bud's surface and c) green filter corresponding to calcein excitation in order to obtain the fluorescent signal's surface. Then the ratio green/red is used to quantify the bud's surface that is irrigated by the calcein fluorescence.

Supplementary Figure 2 Validation of RNA-seq gene expression profiles by qRT-PCR on seven marker genes.

Supplementary Figure 3 Dormancy release and transport capacity characterization. (a) Dormancy release date estimation was done by forcing experiment. (b) Calcein signal profiles recorded in flower buds. Dormancy release date (dotted line).

Supplementary Figure 4 Additional observations of callose accumulation in sweet cherry flower buds. 50 µm sections of flower buds from the 'Fertard' cultivar, were observed with aniline blue fluochrome using an epifluorescent microscope (Zeiss Axiophot).

Supplementary Figure 5 Sampling details for the RNA-seq analysis compared to dormancy status and transport capacity evaluated by the calcein signal (Green to Red fluorescence ratio) to the bud. Flower buds were sampled from the sweet cherry cultivar 'Fertard' during the winter 2017-2018. Stars indicate the sampling dates (Oct 17th 2017, Dec 8th 2017, Jan 3rd 2018, Feb 12th 2018 and Mar 12th 2018). Dotted lines correspond to the dormancy release date estimated as 50% of budbreak after 10 days under forcing conditions.

Supplementary Figure 6 Principal component analysis of samples based on the global gene expression including (a) all Differentially Expressed Genes and (b) transport candidate genes showing the same pattern of dormancy stages.

Supplementary Figure 7 Expression profiles of the differentially expressed genes repressed during endodormancy, classed in clusters 1 and 5. TPM: transcripts per million reads.

Supplementary Figure 8 Expression profiles of the differentially expressed genes activated during ecodormancy, classed in lcusters 2, 3 and 4. TPM: transcripts per million reads.

Supplementary Table 1 Detail information on sequencing and mapping processes for each sample.

Supplementary Table 2 Clustering of differentially expressed genes among 48, 133 genes identified in reference genome, DEGs between each time point which were assessed using DEseq2 R Bioconductor package (Love et al., 2014), in the statistical software R (R Core Team 2022). Genes with an adjusted p-value (padj) < 0.05 and a log fold change > 1 were selected.

Supplementary Table 3 Gene ontology terms obtained using Blast2GO for the sweet cherry 'Regina' reference genome.

Supplementary Table 4 GO enrichment analysis indicating Molecular Functions (MF) and Biological Processes (BP) over-represented in DEGs compared to the entire gene set of reference genome, based on a classic Fisher algorithm.

Supplementary Table 5 Information on the candidate genes evaluated in the study.

Supplementary Table 6 Clustering on the differentially expressed genes among transport candidate genes. DEGs between each time point which were assessed using DEseq2 R Bioconductor package (Love et al., 2014), in the statistical software R (R Core Team 2022). Genes with an adjusted p-value (padj) < 0.05 and a log fold change > 1 were selected.

Supplementary Data Sheet 1 Raw read counts and transcript per million reads (TPM) for each annotated transcript and each sample.

Supplementary Data Sheet 2 DESeq results for all annotated transcripts.
