# Supplementary material for: Transport capacity is uncoupled with endodormancy breaking in sweet cherry buds: physiological and molecular insights
Source: Front Plant Sci. 2023 Nov 14;14:1240642. doi: 10.3389/fpls.2023.1240642 (PMC11094712; doi:10.3389/fpls.2023.1240642)
Supplement: Supplementary Figure 1 — Microscopy observations of flower bud under a) bright light, b) red filter corresponding to chlorophyll excitation in order to obtain the total bud's surface and c) green filter corresponding to calcein excitation in order to obtain the fluorescent signal's surface. Then the ratio green/red is used to quantify the bud's surface that is irrigated by the calcein fluorescence. [file Image_1.pdf]

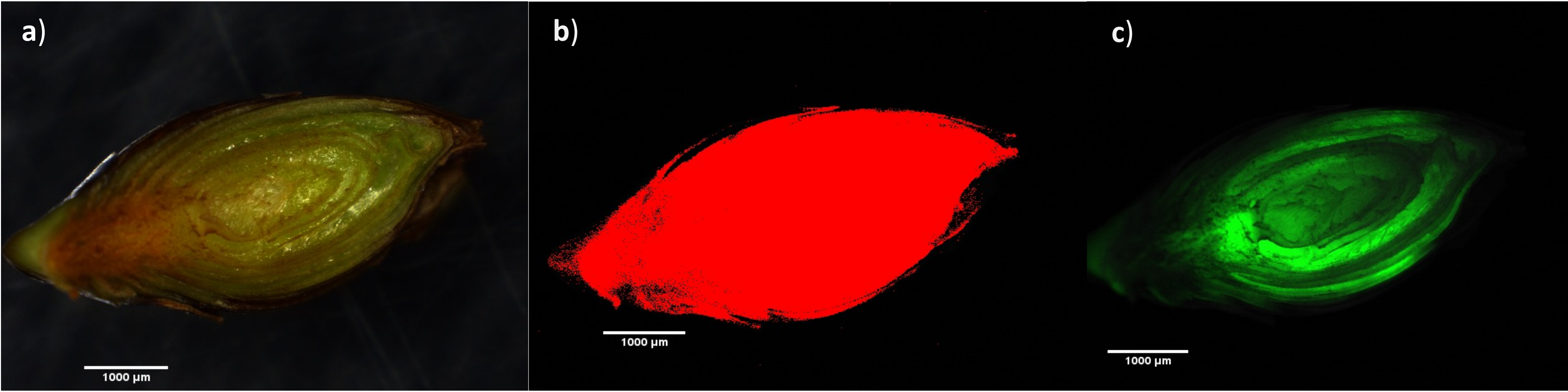

Figure S1. Microscopy observations of flower bud under a) bright light, b) red filter corresponding to chlorophyll excitation in order to obtain the total bud's surface and c) green filter corresponding to calcein excitation in order to obtain the fluorescent signal's surface. Then the ratio green/red is used to quantify the bud's surface that is irrigated by the calcein fluorescence.
