# Supplementary material for: Transport capacity is uncoupled with endodormancy breaking in sweet cherry buds: physiological and molecular insights
Source: Front Plant Sci. 2023 Nov 14;14:1240642. doi: 10.3389/fpls.2023.1240642 (PMC11094712; doi:10.3389/fpls.2023.1240642)
Supplement: Supplementary Figure 2 — Validation of RNA-seq gene expression profiles by qRT-PCR on seven marker genes. [file Image_2.pdf]

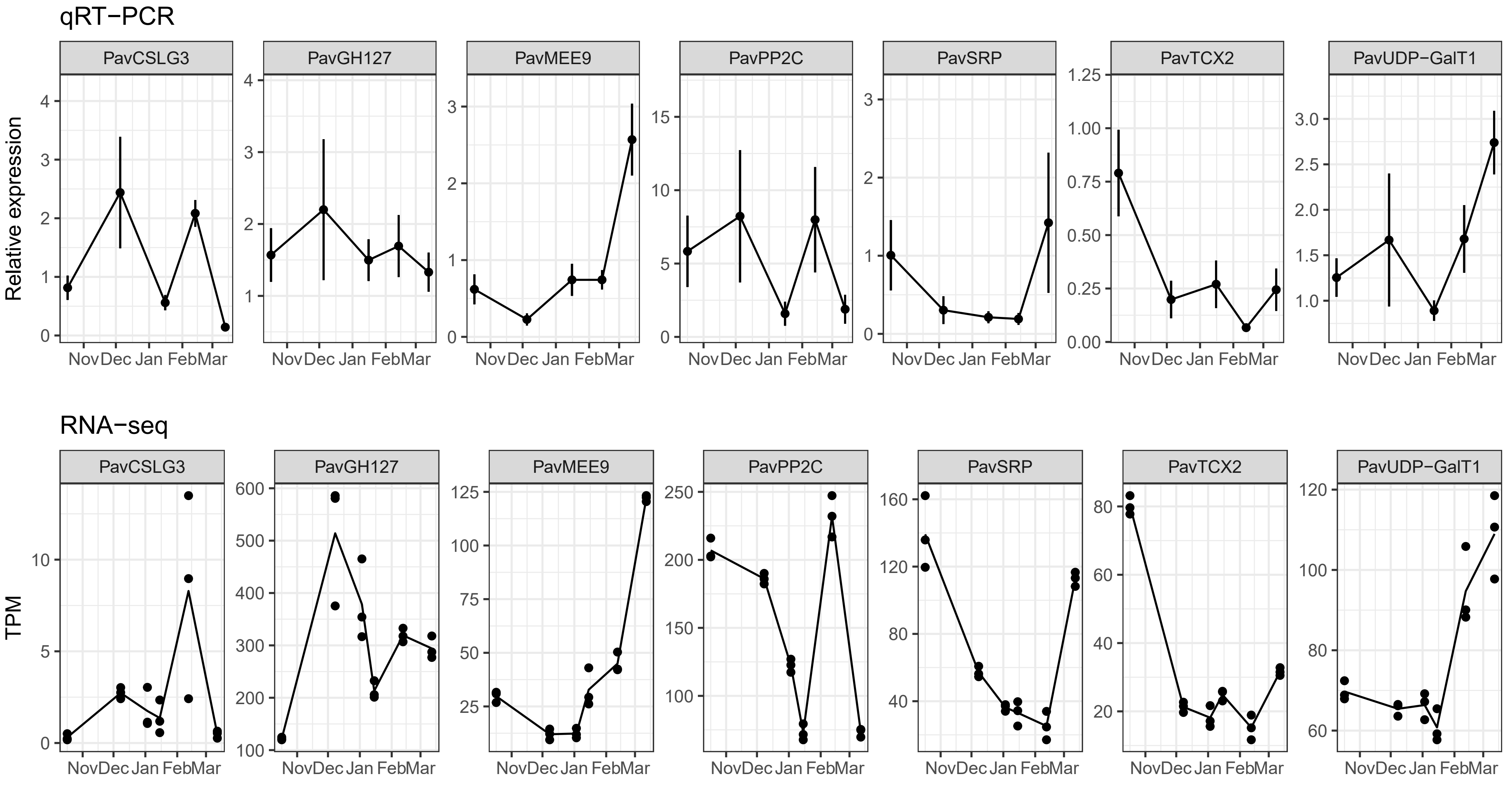

Supplementary Figure 2. Validation of RNA-seq gene expression profiles by qRT-PCR on seven marker genes
