# Supplementary material for: Transport capacity is uncoupled with endodormancy breaking in sweet cherry buds: physiological and molecular insights
Source: Front Plant Sci. 2023 Nov 14;14:1240642. doi: 10.3389/fpls.2023.1240642 (PMC11094712; doi:10.3389/fpls.2023.1240642)
Supplement: Supplementary Figure 3 — Dormancy release and transport capacity characterization. (a) Dormancy release date estimation was done by forcing experiment. (b) Calcein signal profiles recorded in flower buds. Dormancy release date (dotted line). [file Image_3.pdf]

(a)

(b)

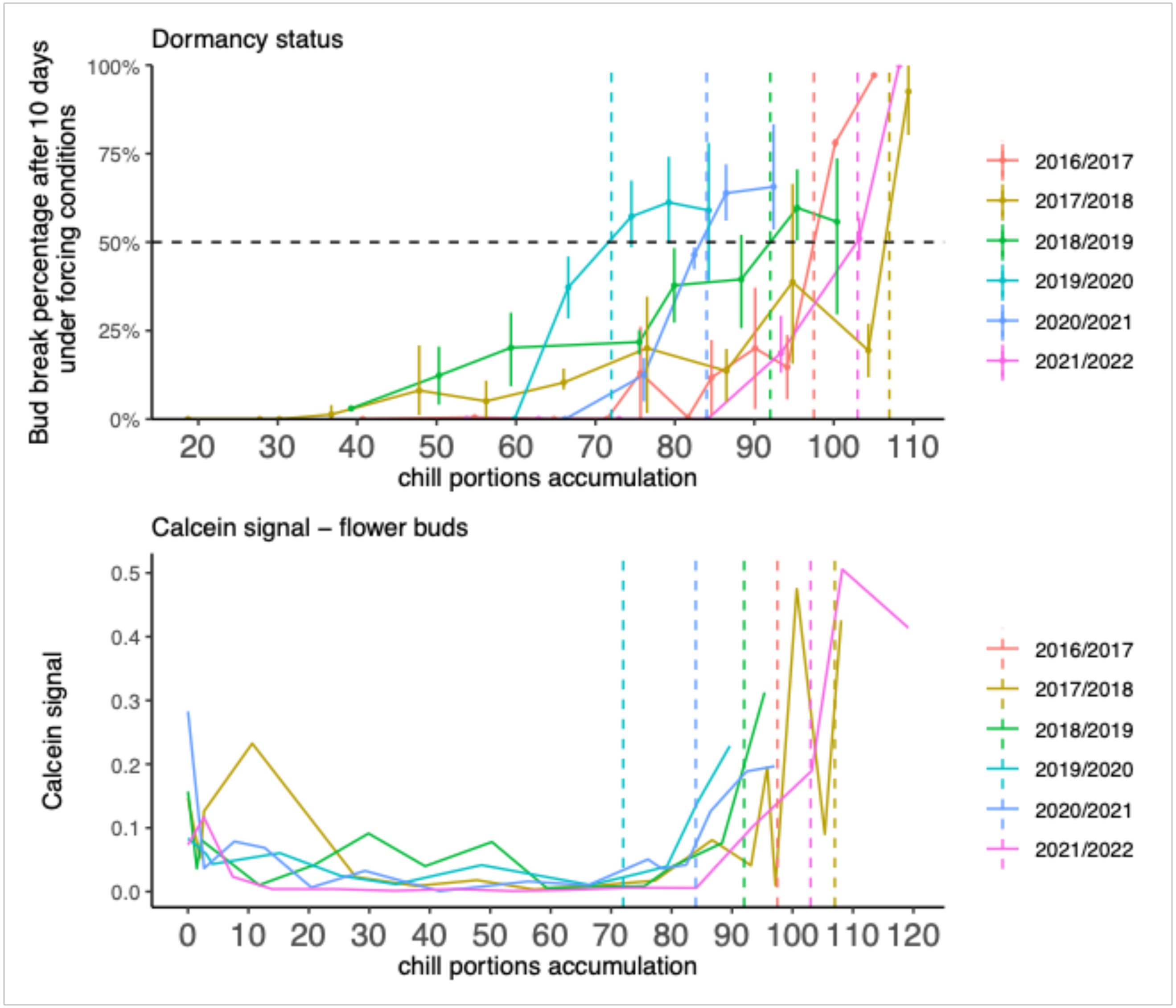

Figure S3. Dormancy release date and transport capacity characterization

a) Dormancy release date estimation was done by forcing experiment

b) Calcein signal profils recorded in flower buds.

Dormancy release date(dotted line).
