# Supplementary material for: Transport capacity is uncoupled with endodormancy breaking in sweet cherry buds: physiological and molecular insights
Source: Front Plant Sci. 2023 Nov 14;14:1240642. doi: 10.3389/fpls.2023.1240642 (PMC11094712; doi:10.3389/fpls.2023.1240642)
Supplement: Supplementary Figure 4 — Additional observations of callose accumulation in sweet cherry flower buds. 50 µm sections of flower buds from the 'Fertard' cultivar, were observed with aniline blue fluochrome using an epifluorescent microscope (Zeiss Axiophot). [file Image_4.pdf]

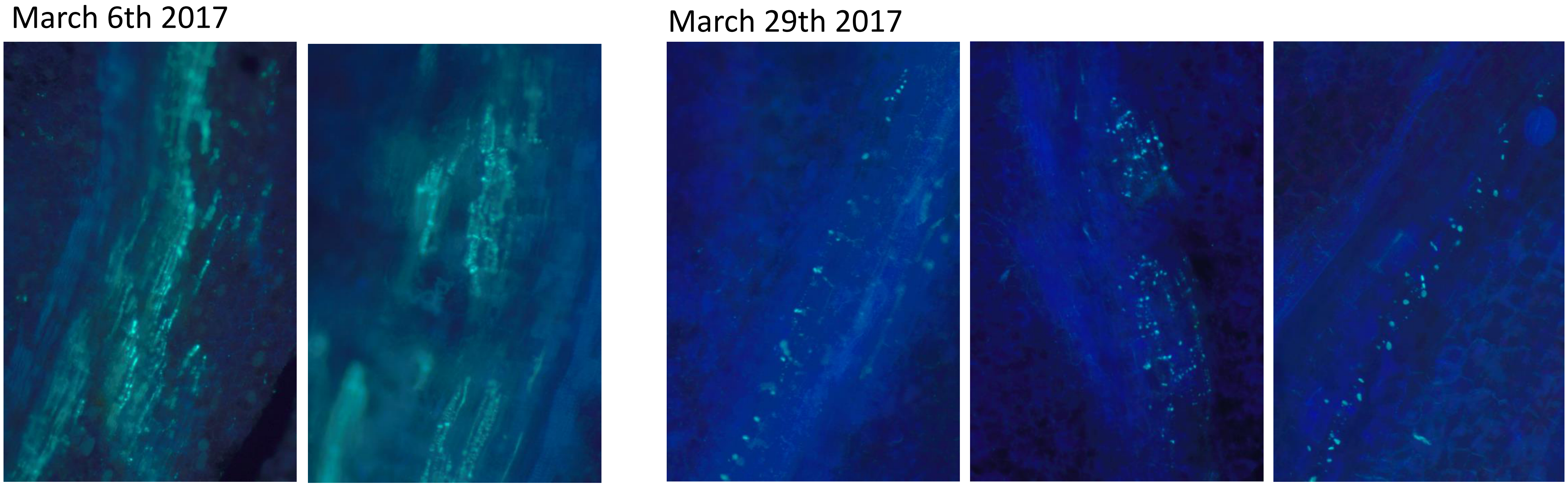

Figure S4. Additionnal observations of callose accumulation in sweet cherry flower buds. 50 µm sections of flower buds from the ‘Fertard’ cultivar, were observed with aniline blue fluochrome using an epifluorescent microscope (Zeiss Axiophot).
