# Supplementary material for: Transport capacity is uncoupled with endodormancy breaking in sweet cherry buds: physiological and molecular insights
Source: Front Plant Sci. 2023 Nov 14;14:1240642. doi: 10.3389/fpls.2023.1240642 (PMC11094712; doi:10.3389/fpls.2023.1240642)
Supplement: Supplementary Figure 5 — Sampling details for the RNA-seq analysis compared to dormancy status and transport capacity evaluated by the calcein signal (Green to Red fluorescence ratio) to the bud. Flower buds were sampled from the sweet cherry cultivar 'Fertard' during the winter 2017-2018. Stars indicate the sampling dates (Oct 17th 2017, Dec 8th 2017, Jan 3rd 2018, Feb 12th 2018 and Mar 12th 2018). Dotted lines correspond to the dormancy release date estimated as 50% of budbreak after 10 days under forcing conditions. [file Image_5.pdf]

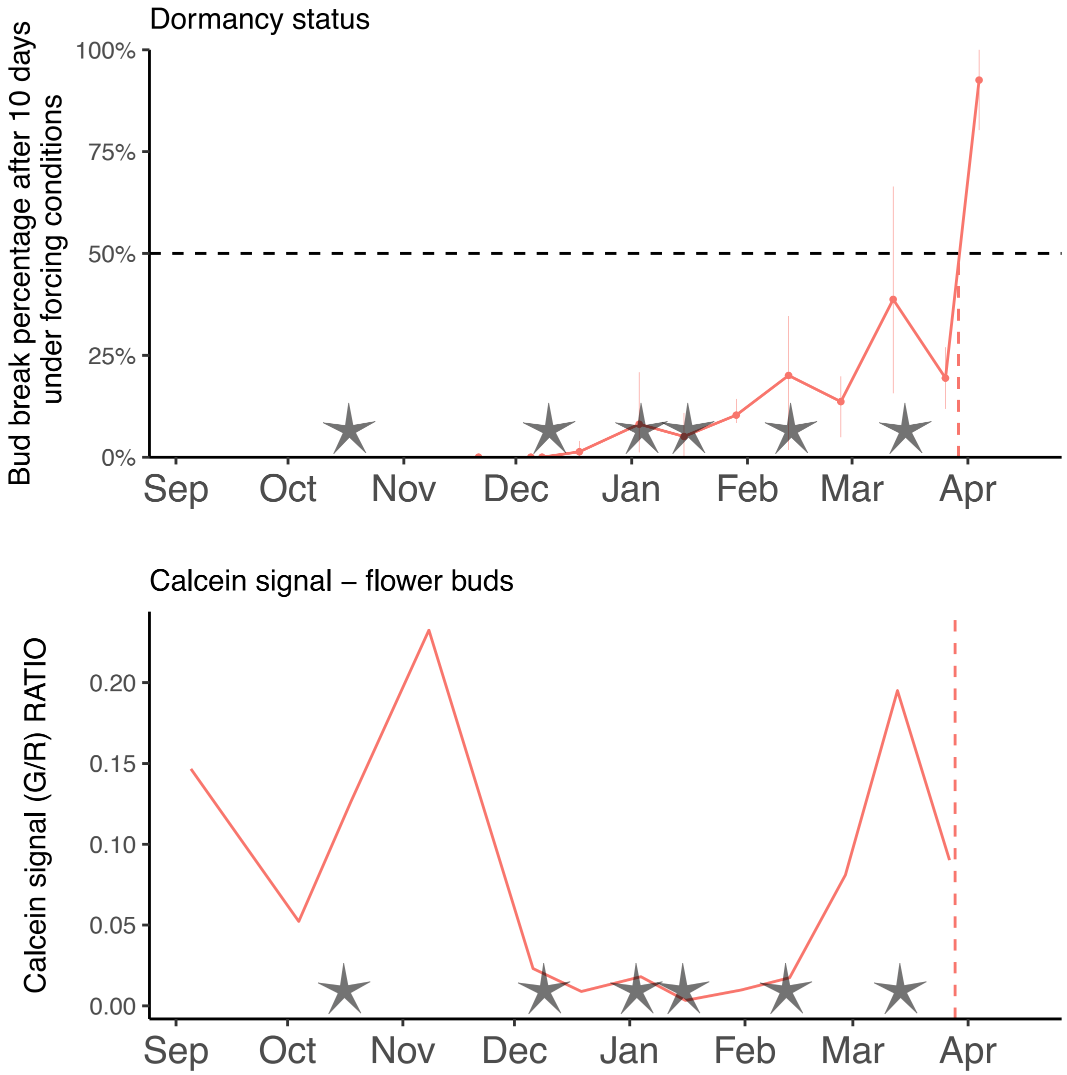

Figure S5. Sampling details for the RNA-seq analysis compared to dormancy status and transport capacity evaluated by the calcein signal (Green to Red fluorescence ratio) to the bud. Flower buds were sampled from the sweet cherry cultivar 'Fertard' during the winter 2017-2018. Stars indicate the sampling dates (Oct 17th 2017, Dec 8th 2017, Jan 3rd 2018, Feb 12th 2018 and Mar 12th 2018). Dotted lines correspond to the dormancy release date estimated as 50% of budbreak after 10 days under forcing conditions.
