# Supplementary material for: Transport capacity is uncoupled with endodormancy breaking in sweet cherry buds: physiological and molecular insights
Source: Front Plant Sci. 2023 Nov 14;14:1240642. doi: 10.3389/fpls.2023.1240642 (PMC11094712; doi:10.3389/fpls.2023.1240642)
Supplement: Supplementary Figure 6 — Principal component analysis of samples based on the global gene expression including (a) all Differentially Expressed Genes and (b) transport candidate genes showing the same pattern of dormancy stages. [file Image_6.pdf]

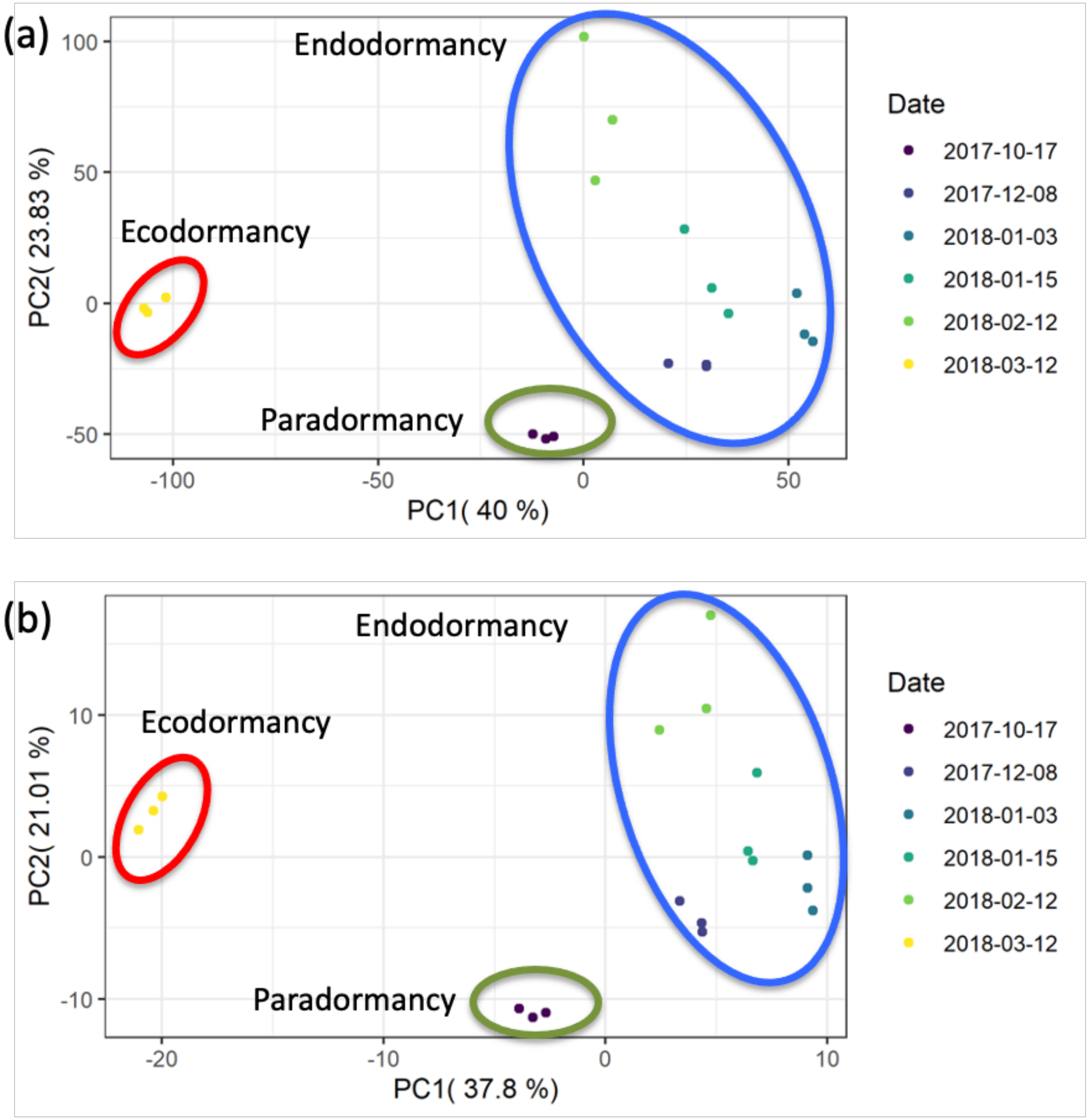

Figure S6. Principale Component Analyses of samples, based on the global genes expression including (a) all Differencially Expressed Genes and (b) transport candidate genes showing the same pattern of dormancy stages.
