# Supplementary material for: Transport capacity is uncoupled with endodormancy breaking in sweet cherry buds: physiological and molecular insights
Source: Front Plant Sci. 2023 Nov 14;14:1240642. doi: 10.3389/fpls.2023.1240642 (PMC11094712; doi:10.3389/fpls.2023.1240642)
Supplement: Supplementary Figure 7 — Expression profiles of the differentially expressed genes repressed during endodormancy, classed in clusters 1 and 5. TPM: transcripts per million reads. [file Image_7.pdf]

Cluster 1

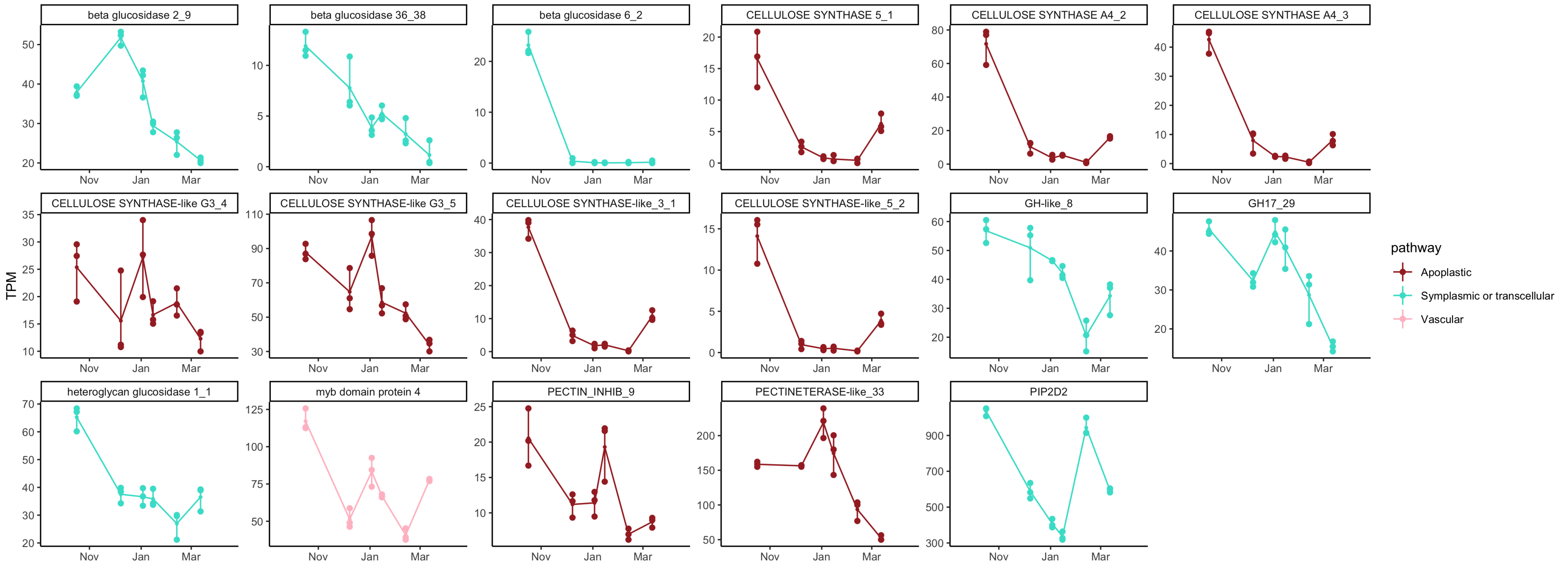

Cluster 5

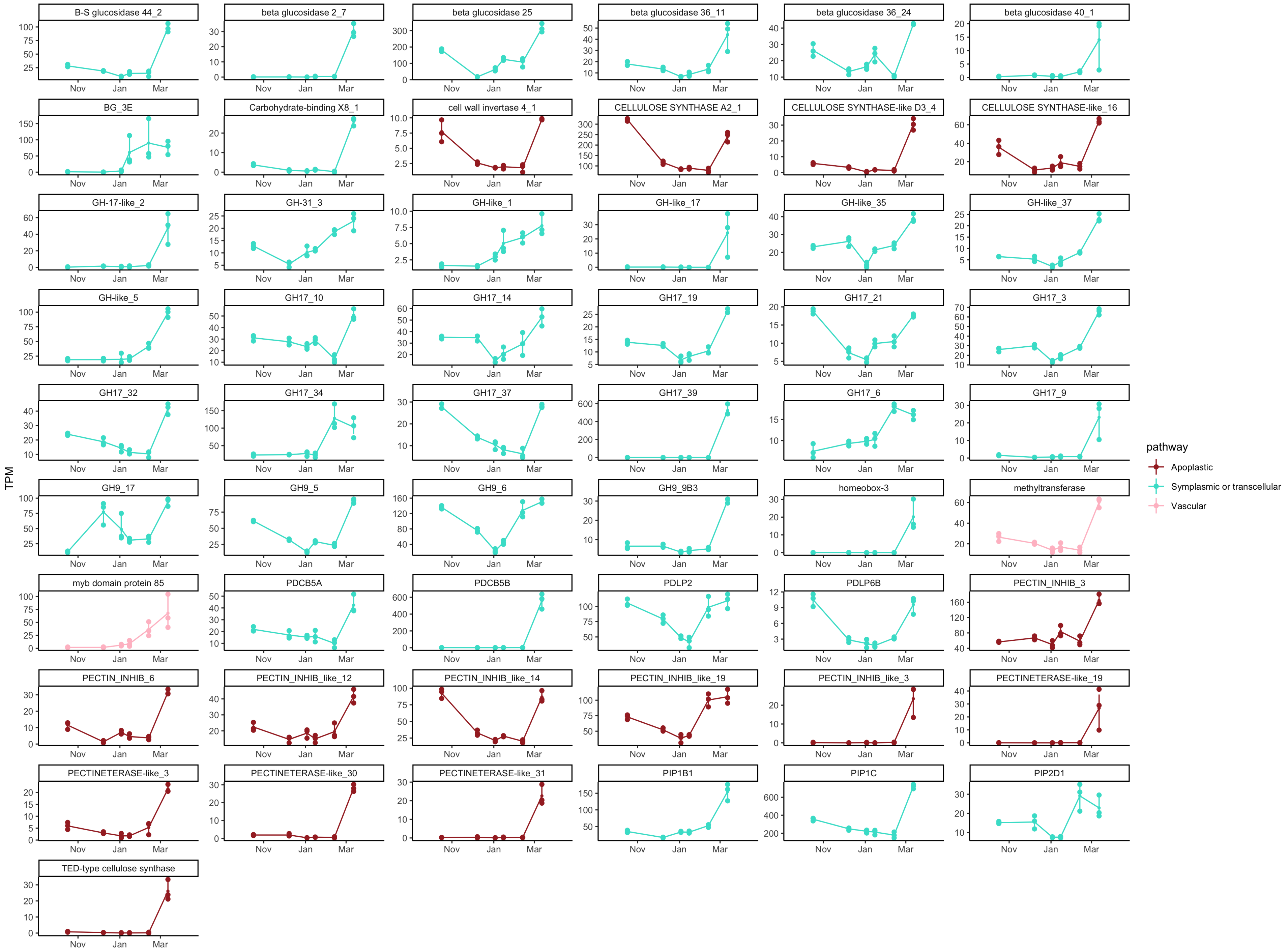

Figure S7. Expression profiles of the differentially expressed genes repressed during endodormancy, classed in clusters 1 and 5. TPM: transcripts per million reads.
