# Supplementary material for: Transport capacity is uncoupled with endodormancy breaking in sweet cherry buds: physiological and molecular insights
Source: Front Plant Sci. 2023 Nov 14;14:1240642. doi: 10.3389/fpls.2023.1240642 (PMC11094712; doi:10.3389/fpls.2023.1240642)
Supplement: Supplementary Figure 8 — Expression profiles of the differentially expressed genes activated during ecodormancy, classed in lcusters 2, 3 and 4. TPM: transcripts per million reads. [file Image_8.pdf]

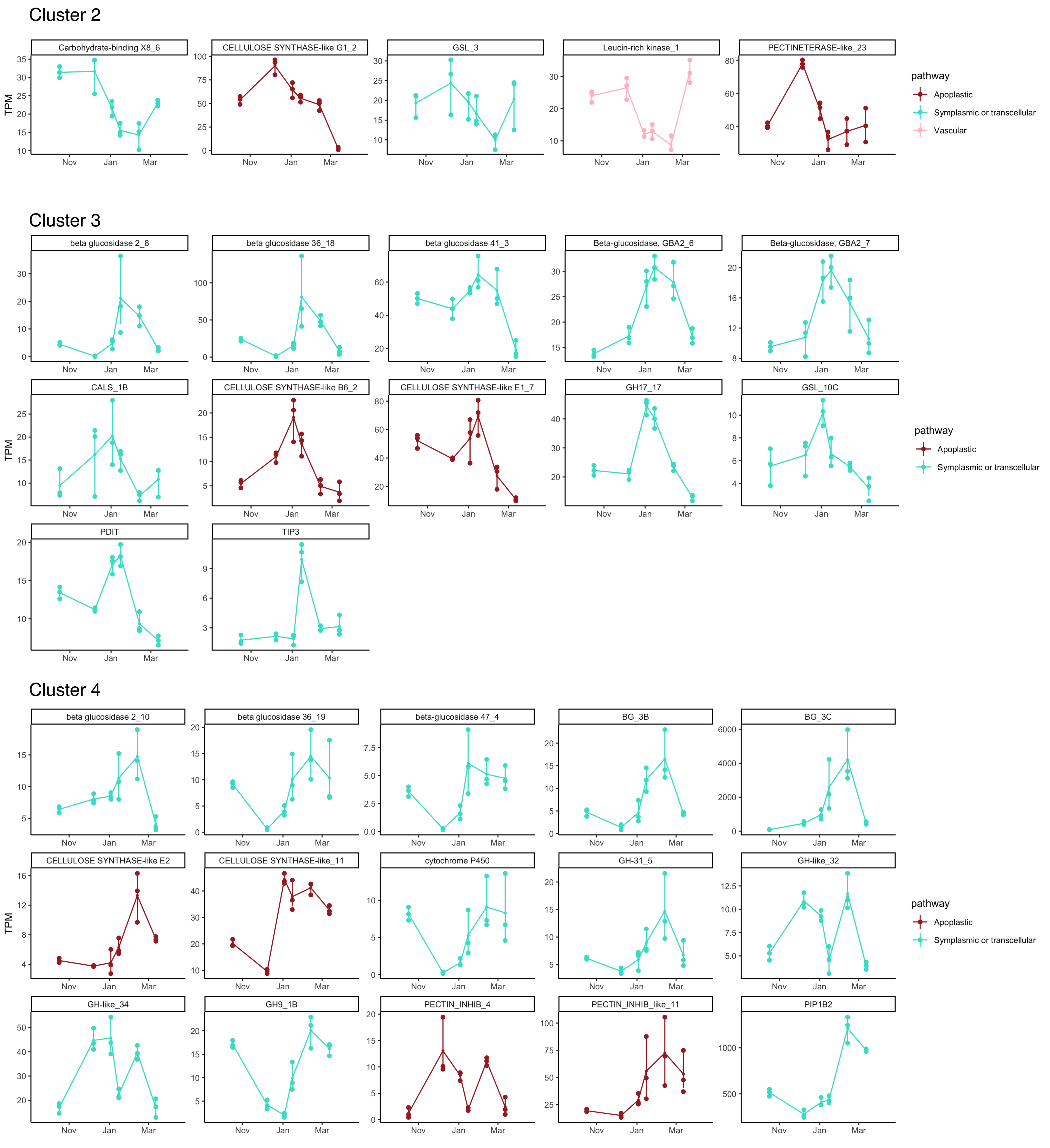

Figure S8. Expression profiles of the differentially expressed genes activated during endodormancy, classed in clusters 2, 3 and 4. TPM: transcripts per million reads.
